# Listen to hard topics with soft ears - domestic violence and family carer; a survey of referrals to a MHIDD forensic mental health service in Ireland

**DOI:** 10.1192/bjo.2021.844

**Published:** 2021-06-18

**Authors:** Mohamed Elhassan Elamin, Anthony Kearns, Aidan Cooney

**Affiliations:** National Forensic Mental Health Services

## Abstract

**Aims:**

A number of studies sought to explore and define families needs, experiences and concerns associated with being a carer for a detained person and their interaction with Forensic services (McKeown et al, 1995, MacInnes et al, 2002, Tsang et al, 2002, Absalom et al, 2012 Horberg et al, 2015).

Relatives can be victims of the service user's offence (Ferriter & Huband, 2003, Tsang et al 2002), and may even blame the service user for their behaviour (Barrowclough et al., 2005). Service user becomes violent and aggressive family members are less likely to be motivated to participate, due to the service user's behaviour (MacInnes, 2000).

An initial domestic violence survey of in-patient case files found that in 66%of the patients files, there were reported incidents of domestic violence in family caring relationships prior to the index offence and subsequent admission to NFMHS (Cooney, 2018).

**Method:**

A quantitative methodology was used. A domestic violence survey of referrals was conducted of 100 referrals to the National Forensic Mental Health Services – Mental Health and Intellectual & Development Disability Services between 2016-2019.

**Result:**

22% of the referrals reported Domestic Violence in the family care-giving relationships.

The father was recorded as the parent to be experience most Domestic Violence; 40%. Other family members who experienced domestic violence ranged from the mother 32%, brother 12% and sister 8%. Other family members were 8%.

100% of the referrals did not report the domestic violence in the carer relationships, nor did referring agencies recorded safeguarding adults concerns.

**Conclusion:**

The findings from this audit raises a couple of clinical, legal and safeguarding adults work in National Forensic Mental Health Services with regards to family work. Firstly, the need to (re)conceptualising family work in the context of trauma informed care. Secondly, family work should offer some families, who are victim of crime, a restorative approach. Thirdly, safeguarding adults will need to consider complex caring relationships and acknowledged this as part of care planning and support.

